# Correction: Toxicity evaluation of gold nanoparticles using an *Allium cepa* bioassay

**DOI:** 10.1039/d6ra90069k

**Published:** 2026-07-10

**Authors:** A. Rajeshwari, S. Suresh, Natarajan Chandrasekaran, Amitava Mukherjee

**Affiliations:** a Centre for Nanobiotechnology, VIT University Vellore 632014 India amit.mookerjea@gmail.com amitav@vit.ac.in +91 416 220 2620

## Abstract

Correction for ‘Toxicity evaluation of gold nanoparticles using an *Allium cepa* bioassay’ by A. Rajeshwari *et al.*, *RSC Adv.*, 2016, **6**, 24000–24009, https://doi.org/10.1039/c6ra04712b.

The authors regret that Fig. 1f in this article was previously published by the authors in another paper without the due citation.^1^ The authors confirm that the materials were the same and reaffirm that the scientific findings and conclusions of the present work remain entirely original and unaffected. The authors would like to state that the 3 nm difference in the mean hydrodynamic diameter between the two research articles (40 ± 1 nm *vs.* 43.11 ± 0.48 nm) falls within the normal variation expected for citrate-reduced gold nanoparticles, and a variation of up to ±5 nm in DLS measurements at different time points for the same gold nanoparticles is plausible and falls within the expected range of instrumental precision.

The corrected Fig. 1 caption should be:

**Fig. 1** The particle size distribution (a–c) and TEM image (d–f) of as-synthesized gold NPs, Au15, Au30, and Au40 [MHD – mean hydrodynamic diameter]. The TEM in Fig. 1f is adopted from previously published work – Fig. 1b in ref. 1.

An independent expert has reviewed this case and the authors' explanation and confirmed that the conclusions of the article are not impacted by this error.

The Royal Society of Chemistry apologises for these errors and any consequent inconvenience to authors and readers.
